# Privacy-Preserving Health Data Collection for Preschool Children

**DOI:** 10.1155/2013/501607

**Published:** 2013-10-29

**Authors:** Shaopeng Guan, Yuan Zhang, Yue Ji

**Affiliations:** ^1^Key Laboratory of Intelligent Information Processing in Universities of Shandong (Shandong Institute of Business and Technology), Yantai 264005, China; ^2^State Key Laboratory for Novel Software Technology, Nanjing University, Nanjing 210093, China; ^3^Preschool Education Department, Nanjing Normal University, Nanjing 210097, China

## Abstract

With the development of network technology, more and more data are transmitted over the network and privacy issues have become a research focus. In this paper, we study the privacy in health data collection of preschool children and present a new identity-based encryption protocol for privacy protection. The background of the protocol is as follows. A physical examination for preschool children is needed every year out of consideration for the children's health. After the examination, data are transmitted through the Internet to the education authorities for analysis. In the process of data collection, it is unnecessary for the education authorities to know the identities of the children. Based on this, we designed a privacy-preserving protocol, which delinks the children's identities from the examination data. Thus, the privacy of the children is preserved during data collection. We present the protocol in detail and prove the correctness of the protocol.

## 1. Introduction

With computers and networks having become an important tool in everyday life, more and more data need to be transmitted through networks. Meanwhile, privacy issues have drawn public attention. How to protect privacy in a network environment has become a research focus in the field of computer network.

Privacy, broadly speaking, refers to private data held by organizations or individuals, which are confidential to others. For individuals, private information such as personal identification, physical condition, and geographical location is all private [1]. The spread of private information will cause a lot of negative consequences, even leading to crimes. Therefore, privacy-preserving technology becomes an important research direction. At present, researches of privacy-protection technology in the network include at least the following areas.

Privacy Protection in Wireless Sensor Networks. Wireless sensor networks have broad application prospects in the fields of environmental monitoring, health care, national defense, and so on. However, in practical applications, wireless sensor networks are facing a serious risk of data disclosure or tampering that will lead to serious consequences [2–5]. For example, in the field of military, data collected by wireless sensor networks often contain important intelligence information which, if disclosed or tampered with, will pose a serious threat or military missteps. The privacy-protecting technology is an indispensable part of wireless sensor networks [6–10].

Privacy Preserving-Data Mining. Data mining is the most important knowledge discovery tool in today's society. It can reveal the hidden rules behind large amounts of data for people. However, data sources used for data mining also contain a lot of individual privacy, business intelligence or government secrets. In the data mining process, if the data are used arbitrarily without any restraint, personal privacy and confidential information will be disclosed, and thereby people's daily lives and even social stability will be seriously affected [11–15]. It is a dilemma to pick up potential and valuable knowledge from the massive amounts of data in data mining and in the meantime preserve privacy. The ideal solution is to transform the raw data, and then prevent the direct and indirect access to private information, while the mining algorithms are still able to get from the converted data almost the same information and knowledge as those from the raw data [16–20]. 

Privacy Studies for Medical and Health Information. In the field of medicine, medical treatments and results must be based on the patients' privacy. With the application of information technology in the medical field, electronic medical records (EMRs) have become the main carrier of medical information. EMRs are prevailing in medical institutions because of their large storage capacity, saving of resources, convenient query, and good sharing of information, which improve the efficiency of diagnosis and treatment [21]. However, since EMRs contain a lot of patients' privacy and are easy to copy and spread, privacy protection is significant in the field of medical and health information [22–24]. 

In addition to the above, as new network applications emerge, some new privacy issues also need to be addressed. For example, in fields such as social network, data publishing, cloud computing, and the Internet of Things, privacy has attracted people's attention [25–28]. In recent years, location privacy in the mobile network also become a highlight as location-based services develop [29, 30].

In this paper, we study the privacy in the health data collection for preschool children and present an identity-based encryption protocol to protect the identities of the children. The background of the protocol is as follows. A physical examination of preschool children is needed every year out of consideration for the children's health. After examination, data need to be transmitted through the Internet to the education authorities for analysis. In the process of data collection, it is unnecessary for the education authorities to know the identities of the children. Based on this, we designed a privacy-preserving protocol, which delinks the children's identities from the examination data. Thus, examination data can be transmitted over the network securely without the disclosure of the children's identities.

The rest of this paper is organized as follows. In Section 2, we briefly review the related works and discuss their relationship with our work. In Section 3, we describe the preliminary and cryptographic tools we use to build our protocol. In Section 4, we present the design of our protocol and analyze it. Finally, we conclude the paper in Section 5.

## 2. Related Works

 With the application of information technology in the field of medicine and health, the privacy issues also begin to grasp people's attention. At present, research on EMRs focuses on three areas: privacy protection of raw data, access control of EMRs, and privacy-protecting medical information system [31–33].

Privacy Protection of Raw Data. Privacy protection of raw data refers to the fact that some technologies such as interference or anonymity are adopted to process raw data and form a new data set before the raw data are provided to others. After transformation, the new data set maintains the same distribution characteristics as the raw data, while it no longer contains personal information and therefore achieves the protection of individual privacy. Most of the existing privacy-protecting technologies of the raw data are based on anonymous method. Anonymizing the raw data will inevitably result in loss of information. Therefore, the research work is focused on finding the tradeoff between the availability of data and privacy protection [34–37]. 

Access Control of EMRs. Using a centralized management of rights, the access control technology is a defensive measure against unauthorized use of data. Its basic objective is to control access rights of users to EMRs or medical information system and thereby ensure that the medical data are used under authorization. Access control is an important measure to protect electronic medical data in information systems, which determines who can access the system and how the data are used. Appropriate access controls can prevent unauthorized users from making accidental or inadvertent access to data; however, the implementation of access control is complex, and the adjustment and management of rights are difficult [38–41].

Privacy-Protecting Medical Information System. In addition to the above privacy-protecting technologies, scholars designed some privacy-protecting medical information systems. In [42], Jieun Song and Myungae Chung put forward a safe framework of health privacy for environmental service model. The system includes authentication, access control and privacy-protecting service. In [43], Gardner and Xiong constructed an identity-conversion system to protect the health information of patients. The system uses *conditional random fields* method to extract identity properties from unstructured data and conduct identity conversion by *k* anonymous method. In [44], Lin et al. proposed a privacy protecting scheme for electronic health systems and proved by formal reasoning that the scheme is able to protect medical privacy and context information simultaneously. However, till now, there is no perfect system architecture of privacy protection. 

On the whole, privacy-protecting technologies in the field of medicine and health have made considerable progress. However, there are still some problems with the existing technologies. For example, security assumptions in the models are too strong to be adopted to the real scenario. In addition, existing privacy-protecting schemes have no universality. Every scheme is only for a specific situation or a specific privacy issue. As far as the privacy in the health data collection for preschool children in this paper is concerned, no existing schemes can be directly adopted. Thus, we design an identity-based encryption protocol for the privacy protection.

## 3. Preliminary

 The identity-based encryption method is a kind of public key cryptosystems. In a public key cryptosystem, a public key of the other party is needed when users send encrypted messages or verify a digital signature. In order to ensure the legitimacy of the public key, the traditional public key cryptosystem adopts a public key infrastructure, in which a trusted party, called the certification authority (CA), is responsible for authenticating and issuing the corresponding public key certificate of users. The public key certificate binds the identity of a user with its public key. In this kind of system, the CA is responsible for the generation, issuance, storage, maintenance, and withdrawal of public key certificates for users, which requires a significant amount of computing and storage resources.

In 1984, an identity-based encryption (IBE) scheme was presented by Shamir [45], which simplified the management of public key certificates in traditional public key infrastructures. The IBE method directly adopts a user's identity information as the public key. The private key is generated by private key generators (PKGs). Therefore, the communicating parties can take each other's identity as public keys for communication encryption, without the need to get special public key certificates and authenticate the identities. The IBE method no longer needs the support of the CA, avoiding the establishment and management of public key infrastructure in traditional public key cryptography system. In 2000, Sakai, Ohgishi, and Kasahara suggested that bilinear maps on elliptic curves can be used to design the identity-based cryptography scheme. In 2001, Boneh and Franklin realized the first practical IBE scheme using bilinear maps on elliptic curves and proved that the scheme is resistant to chosen-ciphertext attacks in the random oracle model [46]. Since then, the bilinear maps on elliptic curves have gradually become the main tool of identity-based cryptography scheme. 

In applications, the IBE scheme is typically composed of four algorithms [47].

- *Setup*. Select a security parameter *k*, and get system parameters (*params*) and the master key. *Params* include a limited message space *M* and a limited ciphertext space *C*, which are open. The master key is private to PKG.
- *Extract*. Input *params*, the master key, and ID ∈ {0,l}*, and get the private key *d*. ID is an arbitrary sequence as a public key; *d* is the corresponding private key. The Extract algorithm is used to extract private keys from given public keys.
- *Encrypt*. Input params, ID, and *M*_0_ ∈ *M*, and get the ciphertext *C*_0_ ∈ *C*.
- *Decrypt*. Input params, *C*_0_ ∈ *C*, and the private key *d*, and obtain *M*_0_ ∈ *M*.

The above algorithms must be consistent. That is to say, for a given ID, when the private key *d* is extracted by the Extract algorithm, there is Decrypt (*params*, *C*_0_, *d*) = *M*_0_, where *M*_0_ ∈ *M* and *C*_0_ = Encrypt (*params*, ID, *M*_0_). 

Based on bilinear maps on elliptic curves, we design our IBE scheme in this paper which slightly differs from the Boneh-Franklin cryptosystem but is equivalent in terms of security. It consists of four algorithms as follows.

*Initialization.* Let *k* be a security parameter and *q* be a *k*-bit prime. Suppose *G*_1_ and *G*_2_ are two cyclic groups of prime order *q* and $\hat{e}:G_{1}\times G_{1}\to G_{2}$ is an admissible bilinear map with generator *P* of group *G*_1_. (See [46] for the definition of admissible bilinear maps). Assume that identities are *L*-bit strings (where *L* is polynomial in *k*). Consider a cryptographic hash function *H* : {0,1}^*L*^ → *G*_1_. The public key generator (PKG) chooses *s* ∈ {0, 1, …, *q* − 1} uniformly at random and computes *P*_pub_ = *sP*. Here *s* is the *master private key*, while all other parameters mentioned above are public.

*Private Key Generation.* For an identity ID, the private key is *x*_ID_ = *sQ*_ID_ and the public key is *Q*_ID_ = *H*(ID).

*Encryption.* To encrypt *d* ∈ *G*_2_ under identity ID, one can compute $E_{\text{ID}}(d,r)=(d\cdot \hat{e}(P_{\text{pub}},rQ_{\text{ID}}),rP)$, where *r* ∈ {0, 1, …, *q* − 1} is picked uniformly at random. 

*Decryption.* Let *C*  ≝  (*C* ^(1)^, *C* ^(2)^) be a valid ciphertext under identity ID. Then, *C* can be decrypted as follows:

(1) $$\begin{array}{c} D_{x_{\text{ID}}}\left(C\right)=\frac{C^{\left(1\right)}}{\hat{e}\left(x_{\text{ID}},C^{\left(2\right)}\right)}. \end{array}$$

The protocol is homomorphic. That is to say, the following equation is satisfied:

(2) $$\begin{array}{c} E_{\text{ID}}\left(d_{1}d_{2},r_{1}+r_{2}\right)=E_{\text{ID}}\left(d_{1},r_{1}\right)E_{\text{ID}}\left(d_{2},r_{2}\right), \end{array}$$

where the product of the two ciphertexts is defined as taking the product of each component of the ciphertexts.

## 4. Protocol Design and Analysis

Suppose ID_*A*_ is the identity of the preschool child and ID_*B*_ is the identity of a volunteer helper (who could be one of the preschool children volunteering to contribute his computational resources). We assume the administrator does not collude with the volunteer helper (if there is a risk of collusion, we can extend this protocol by adding more helpers, which is straightforward).

Let *n* be the total number of preschool children. Assume that, before the health data are transmitted, each preschool child *i* has been assigned a unique number *R*_*i*_ ∈ {1, 2, …, *n*}, such that no two preschool children have the same number, that is, for any *i* ≠ *j*, *R*_*i*_ ≠ *R*_*j*_.

The protocol includes two phases: a health data submission phase and a decryption phase.

In the *i*th round of the health data submission phase, each preschool child *j* first compares his own number *R*_*j*_ with *i*. If *R*_*j*_ = *i*, then he submits.

(3) $$\begin{array}{c} D_{i,j}=E_{{\text{ID}}_{A}+{\text{ID}}_{B}}\left(d_{j},r_{i,j}\right), \end{array}$$

where *d*_*j*_ is his health data and *r*_*i*,*j*_ is picked uniformly at random. If *R*_*j*_ ≠ *i*, then he submits

(4) $$\begin{array}{c} D_{i,j}=E_{{\text{ID}}_{A}+{\text{ID}}_{B}}\left(1,r_{i,j}\right), \end{array}$$

where *r*_*i*,*j*_ is also picked uniformly at random.

Upon receiving the encryptions in the *i*th round of the health data submission phase, the administrator computes

(5) $$\begin{array}{c} D_{i}=\overset{n}{\underset{j=1}{\prod }}D_{i,j}. \end{array}$$

In the decryption phase, the administrator first forwards all *D*_*i*_ to the helper, who computes

(6) $$\begin{array}{c} {\hat{d}}_{i}=D_{x_{\text{I}{\text{D}}_{A}}}\left(D_{i}\right) \end{array}$$

and returns it to the administrator. Suppose *D*_*i*_ = (*D*_*i*_^(1)^, *D*_*i*_^(2)^). Then, the administrator computes

(7) $$\begin{array}{c} {\overset{\sim }{d}}_{i}=D_{x_{\text{I}{\text{D}}_{A}}}\left({\hat{d}}_{i},D_{i}^{\left(2\right)}\right). \end{array}$$

**Theorem 1**

The protocol is correct; that is, assuming all involved parties follow the protocol, then $\left({\overset{\sim }{d}}_{1},{\overset{\sim }{d}}_{2},\ldots ,{\overset{\sim }{d}}_{n}\right)$ is a permutation of (*d*_1_, *d*_2_, …, *d*_*n*_).

**Proof**

It is easy to see that, assuming all involved parties follow the protocol,

(8) d~i=DxIDA(d^i,Di(2))=d^ie^(xIDA,Di(2))=DxIDB(Di)e^(xIDA,Di(2))=Die^(xIDA,Di(2))e^(xIDB,Di(2)).

Let *J*(*i*) be the value of *j* such that *R*_*j*_ = *i*. Hence,

(9) d~i=Die^(xIDA,Di(2))e^(xIDB,Di(2))=∏j=1nDi,je^(xIDA,Di(2))e^(xIDB,Di(2))=EIDA+IDB(dJ(i),ri,j)∏j≠J(i)EIDA+IDB(1,ri,j)  e^(xIDA,Di(2))e^(xIDB,Di(2))=dJ(i).

Since *J*(*i*) is a permutation on (1,2,…, *n*), the result is a permutation of (*d*_1_, *d*_2_,…, *d*_*n*_).

## 5. Conclusion 

With the development of network technology, more and more data need to be transmitted over the network. Related privacy issues also become a hot research topic. We studied the privacy issue of health data transmitted over the network. For the sake of children's health, a physical examination of preschool children is needed every year. The data need to be transmitted to the education authorities over the Internet for health analysis after examination. In the process of data collection, it is unnecessary for the education authorities to know the identities of the children. Therefore, we designed a privacy-preserving protocol for health data transmission, which delinks the children's identities from the examination data. The protocol is composed of three algorithms: Setup, Encrypt, and Decrypt. At last, we proved the correctness of the protocol.

## References

[1] Agrawal R, Srikant R (2000). Privacy-preserving data mining. *SIGMOD Record (ACM Special Interest Group on Management of Data)*.

[2] Bista R, Chang J-W (2010). Privacy-preserving data aggregation protocols for wireless sensor networks: a survey. *Sensors*.

[3] Tan CC, Wang H, Zhong S, Li Q (2009). IBE-lite: a lightweight identity-based cryptography for body sensor networks. *IEEE Transactions on Information Technology in Biomedicine*.

[4] Carbunar B, Yu Y, Shi W, Pearce M, Vasudevan V (2010). Query privacy in wireless sensor networks. *ACM Transactions on Sensor Networks*.

[5] Zhong S (2008). Efficient, anonymous, and authenticated conference key setup in cellular wireless networks. *Computers and Electrical Engineering*.

[6] Chow C-Y, Mokbel MF, He T (2011). A privacy-preserving location monitoring system for wireless sensor networks. *IEEE Transactions on Mobile Computing*.

[7] Li N, Zhang N, Das SK, Thuraisingham B (2009). Privacy preservation in wireless sensor networks: a state-of-the-art survey. *Ad Hoc Networks*.

[8] Zhong S, Wu F (2010). A collusion-resistant routing scheme for noncooperative wireless Ad Hoc networks. *IEEE/ACM Transactions on Networking*.

[9] Jose J, Princy M, Jose J (2013). Integrity protecting and privacy preserving data aggregation protocols in wireless sensor networks: a survey. *International Journal of Computer Network and Information Security*.

[10] Zhong S, Wu F On designing collusion-resistant routing schemes for non-cooperative wireless ad hoc networks.

[11] Bogdanov D, Jagomägis R, Laur S (2012). *A Universal Toolkit for Cryptographically Secure Privacy-Preserving Data Mining*.

[12] Gurevich A, Gudes E Privacy preserving data mining algorithms without the use of secure computation or perturbation.

[13] Li Y, Chen M, Li Q, Zhang W (2012). Enabling multilevel trust in privacy preserving data mining. *IEEE Transactions on Knowledge and Data Engineering*.

[14] Shah D, Zhong S (2007). Two methods for privacy preserving data mining with malicious participants. *Information Sciences*.

[15] Vaidya J, Zhu YM, Clifton CW (2006). *Privacy Preserving Data Mining*.

[16] Abou-el-ela Abdou Hussien N, Hamza HA (2013). Attacks on anonymization-based privacy-preserving: a survey for data mining and data publishing. *Journal of Information Security*.

[17] Hao Z, Zhong S, Yu N (2011). A privacy-preserving remote data integrity checking protocol with data dynamics and public verifiability. *IEEE Transactions on Knowledge and Data Engineering*.

[18] Aggarwal CC, Philip SY (2008). *A General Survey of Privacy-Preserving Data Mining Models and Algorithms*.

[19] Chen T, Zhong S (2009). Privacy-preserving backpropagation neural network learning. *IEEE Transactions on Neural Networks*.

[20] Zhong S (2007). Privacy-preserving algorithms for distributed mining of frequent itemsets. *Information Sciences*.

[21] Chen L, Yang JJ, Wang Q Privacy-preserving data publishing for free text Chinese electronic medical records.

[22] Goldberg IV (2000). Electronic medical records and patient privacy. *The Health Care Manager*.

[23] Haas S, Wohlgemuth S, Echizen I, Sonehara N, Müller G (2011). Aspects of privacy for electronic health records. *International Journal of Medical Informatics*.

[24] Miller AR, Tucker C (2009). Privacy protection and technology diffusion: the case of electronic medical records. *Management Science*.

[25] Hao Z, Zhong S, Yu N (2011). A time-bound ticket-based mutual authentication scheme for cloud computing. *International Journal of Computers, Communications and Control*.

[26] Oleshchuk V Internet of things and privacy preserving technologies.

[27] Fung BCM, Wang K, Chen R, Yu PS (2010). Privacy-preserving data publishing: a survey of recent developments. *ACM Computing Surveys*.

[28] Zhou B, Pei J, Luk W (2008). brief survey on anonymization techniques for privacy preserving publishing of social network data. *ACM SIGKDD Explorations Newsletter*.

[29] Krumm J (2009). A survey of computational location privacy. *Personal and Ubiquitous Computing*.

[30] Magkos E (2011). Cryptographic approaches for privacy preservation in location-based services: a survey. *International Journal of Information Technologies and Systems Approach*.

[31] Buckovich SA, Rippen HE, Rozen MJ (1999). Driving toward guiding principles: a goal for privacy, confidentiality, and security of health information. *Journal of the American Medical Informatics Association*.

[32] Diaz JA, Griffith RA, Ng JJ, Reinert SE, Friedmann PD, Moulton AW (2002). Patients’ use of the internet for medical information. *Journal of General Internal Medicine*.

[33] Damschroder LJ, Pritts JL, Neblo MA, Kalarickal RJ, Creswell JW, Hayward RA (2007). Patients, privacy and trust: Patients’ willingness to allow researchers to access their medical records. *Social Science and Medicine*.

[34] Zhong S, Yang Z, Wright RN Privacy-enhancing k-anonymization of customer data.

[35] Agrawal R, Johnson C (2007). Securing electronic health records without impeding the flow of information. *International Journal of Medical Informatics*.

[36] Szarvas G, Farkas R, Busa-Fekete R (2007). State-of-the-art anonymization of medical records using an iterative machine learning framework. *Journal of the American Medical Informatics Association*.

[37] Loukides G, Gkoulalas-Divanis A, Malin B (2010). Anonymization of electronic medical records for validating genome-wide association studies. *Proceedings of the National Academy of Sciences of the United States of America*.

[38] Chen T-S, Liu C-H, Chen T-L, Chen C-S, Bau J-G, Lin T-C (2012). Secure Dynamic access control scheme of PHR in cloud computing. *Journal of Medical Systems*.

[39] Ferreira A, Cruz-Correia R, Chadwick D, Antunes L Improving the implementation of access control in EMR.

[40] Gunti N, Sun W, Xu M, Liu Z, Niamat M, Alam M (2012). A healthcare information system with augmented access controls. *Web Technologies and Applications*.

[41] Martino LD, Ni Q, Lin D, Bertino E Multi-domain and privacy-aware role based access control in eHealth.

[42] Jieun S, Myungae C SHOES: secure healthcare oriented environement service model.

[43] Gardner J, Xiong L HIDE: an integrated system for health information DE-identification.

[44] Lin X, Lu R, Shen X, Nemoto Y, Kato N (2009). Sage: a strong privacy-preserving scheme against global eavesdropping for ehealth systems. *IEEE Journal on Selected Areas in Communications*.

[45] Shamir A (1985). *Identity-Based Cryptosystems and Signature Schemes. Advances in Cryptology*.

[46] Boneh D, Franklin M (2001). *Identity-Based Encryption from the Weil Pairing. Advances in Cryptology-CRYPTO 2001*.

[47] Chatterjee S, Sarkar P (2011). *Identity-Based Encryption*.

